# Integrated vector control of *Aedes aegypti* mosquitoes around target houses

**DOI:** 10.1186/s13071-017-2596-4

**Published:** 2018-02-08

**Authors:** Roberto Barrera, Manuel Amador, Jorge Munoz, Veronica Acevedo

**Affiliations:** 1 Entomology and Ecology Activity, Dengue Branch, Centers for Disease Control and Prevention, 1324 Calle Canada, San Juan, 00920 Puerto Rico; 2 Molecular Diagnostic Laboratory, Dengue Branch, Centers for Disease Control and Prevention, 1324 Calle Canada, San Juan, 00920 Puerto Rico

**Keywords:** *Aedes aegypti*, Zika, Vector control, AGO traps, Mosquitoes

## Abstract

**Background:**

The developing fetuses of pregnant women are at high risk of developing serious birth defects following Zika virus infections. We applied an Integrated Vector Control (IVC) approach using source reduction, larviciding, and mass trapping with non-insecticidal sticky traps to protect targeted houses by reducing the density of female *Aedes aegypti* mosquitoes.

**Methods:**

We tested the hypothesis that *Ae. aegypti* density could be reduced to below three female mosquitoes/trap/week around a target house in the center of a circular area with a 150 m radius using IVC. Two non-adjacent areas within the same neighbourhood were selected and randomly designated as the treatment or control areas. Sentinel Autocidal Gravid Ovitraps (SAGO traps) were placed in each study area and were sampled weekly from May to November, during the 2016 Zika epidemic in Puerto Rico. The experimental design was longitudinal with pre-and post-IVC treatment observations between treatment and control areas, and a partial cross-over design, where IVC was applied to the original control area after 2 months to determine if *Ae. aegypti* density converged to levels observed in the treatment area. Pools of female *Ae. aegypti* mosquitoes were analyzed by RT-PCR to detect Zika, dengue and chikungunya virus RNA.

**Results:**

Overall, pre-treatment mosquito densities in the inner (0–50 m; 15.6 mosquitoes/trap/week), intermediate (50–100 m; 18.1) and outer rings (100–150 m; 15.6) were reduced after treatment to 2.8, 4.1, and 4.3 in the inner, middle, and outer rings, respectively. Density at the target house in the treatment area changed from 27.7 mosquitoes/trap/week before IVC to 2.1 after IVC (92.4% reduction), whereas after treating the original control area (cross-over) density changed from 22.4 to 3.5 (84.3% reduction). Vector reductions were sustained in both areas after IVC. Zika virus was detected in *Ae. aegypti*, but the low incidence of the virus precluded assessing the impact of IVC on Zika transmission during the study.

**Conclusions:**

Applying IVC to circular areas that were surrounded by untreated areas significantly decreased the number of mosquitoes around target houses located in the center. Gravid *Ae. aegypti* females in the center of the 150 m areas fell below threshold levels that possibly protect against novel invading arboviruses, such as chikungunya and Zika.

## Background

The recent invasion of Zika virus (ZIKV; *Flaviviridae*; *Flavivirus*) in the Americas [1] has revealed unusual health impacts not previously seen following infections by related arboviruses, such as congenital ZIKV infection syndrome [2] and Guillain-Barré syndrome [3]. Also unusual for an arbovirus is that ZIKV can be transmitted by sexual intercourse [4]. The primary mode of ZIKV transmission is through the bite of infected *Aedes* spp. mosquitoes (Diptera: Culicidae). Developing fetuses of Zika infected pregnant women are at risk for serious birth defects, while most non-pregnant symptomatic persons do not have life threatening disease [5]. The majority of ZIKV infections are asymptomatic [6], making case detection and control more difficult.

Because of the greater risk ZIKV poses to pregnant women, public health agencies have recommended that pregnant women and women attempting pregnancy do not travel to areas with ongoing transmission and to protect themselves against mosquito bites [7]. Protection from mosquito bites include cleaning patios and gardens to eliminate standing water and the use of mosquito repellents, long sleeve clothing, socks, closed shoes, screens in windows and doors, and air conditioning [7]. One question of interest is what sort of focal or local vector control measures can be implemented to protect pregnant women at home, work, or other sites where pregnant women spend significant amount of time.

In the case of dengue virus, focal control of *Ae. aegypti* is typically conducted at the home of a dengue case and in neighbouring homes to contain the spread of the virus [8]. The treated area is chosen so that it has a radius representing the average flight distance of female *Ae. aegypti*. The flight distance of *Ae. aegypti* is a few hundred meters [9] and varies depending on the physiological state of the released mosquitoes (unfed, recently fed, gravid), abundance of aquatic habitats and vertebrate hosts, and landscape [10]. Selection of the treatment area also takes into account the practicality of the vector control intervention, because increasing the radius implies increasing the number of houses to be treated in a quadratic fashion. For example, if an average house occupies 100 m^2^, doubling the radius from 100 to 200 m would quadruple the number of houses to be treated, from 314 to 1256 houses.

The objective of this investigation was to test if Integrated Vector Control (IVC) using a combination of source reduction, larviciding, and Autocidal Gravid Ovitraps (AGO traps) [11] applied to at least 80% of houses in an area with a 150 m radius significantly reduced the density of *Ae. aegypti* around a pre-selected house in its center. The main hypothesis was that vector control would be most effective at the center of the area (around and at the chosen house) because it is the farthest place from nearby infested areas (outside the 150 m radius treated area) from where dispersing *Ae. aegypti* females could fly into without being caught in the traps. A previous study in the same municipality and under similar vector control treatments showed that people living in neighbourhoods with less than three female *Ae. aegypti* per AGO trap per week had 50 % lower incidence of chikungunya antibody [12] and ten times lower incidence of positive pools of *Ae. aegypti* [13]. Therefore, we considered a significant reduction of female *Ae. aegypti* in this study to exist if mosquito density fell to or below that threshold.

## Methods

**Fig. 1:**
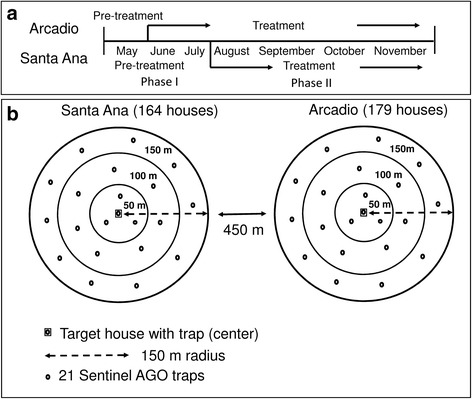
Diagram of experimental design. **a** Timelines: Pre-intervention (May 2016) and Intervention (June–November 2016) periods in Arcadio and Pre-intervention (May–July 2016) and Intervention (July–November 2016) periods in Santa Ana. **b** Buffers 150 m around target houses in Santa Ana and Arcadio, showing the approximate locations of 21 surveillance AGO traps and 50 m rings around target houses

The study was conducted in El Coco (5758 inhabitants; 2353 houses; US Census 2010), which is located in Salinas Municipality (18°00′00"N; 66°15′20"W), southern Puerto Rico. Most dwellings are one-story buildings with patios or gardens, typical of most urban areas in Puerto Rico. Two circular areas of 150 m radius each were selected in the neighbourhood so that they had similar housing conditions and density but were not adjacent (450 m apart). The two selected sectors were Santa Ana and Arcadio. Santa Ana was designated at random to be the “control area” and Arcadio the “IVC treated area”. The treatment consisted of applying source reduction and larviciding, and placing three traps per home, as explained below. The 150 m buffer area around a house at the center can be thought of having three 50 m rings: the inner 0–50 m, middle 50–100 m, and outer rings (100–150 m; Fig. 1). Twenty one sentinel AGO (SAGO) traps were deployed in each circular area, with four, seven, and ten traps located in the inner, middle, and outer rings, respectively (Fig. 1). One of the four traps in the inner ring was purposely located at the chosen house. The Santa Ana study site had 164 houses and Arcadio 179 houses. SAGO traps were placed at the beginning of May until the end of the study in November 2016 in both study areas. The same AGO trap design was used for both surveillance control purposes, the difference was that sentinel traps (SAGO) were checked every week to enumerate mosquitoes, whereas the AGO traps used for control purposes were not.

The experimental design included two phases: (i) a pre-treatment period of 3 weeks in May 2016, followed by IVC in Arcadio to compare *Ae. aegypti* density with the untreated Santa Ana until July 2016; and (ii) Santa Anna was treated with IVC after July 2016 to determine if IVC produced the same effects observed in Arcadio (initial treatment area). This is an incomplete cross-over design because we did not cease controlling *Ae. aegypti* in Arcadio due to the ongoing Zika epidemic, and therefore we did not investigate what would have happened if vector control was lifted (Fig. 1a). Integrated Vector Control consisted of eliminating, cleaning or modifying containers (source reduction), applying larvicide (Altosid Pro-G), and placement of three AGO traps in the backyards of most houses (84%). Source reduction and larviciding were applied only once, whereas AGO traps were kept in place for the duration of the study after placement. Only two control AGO traps were added to houses already having one SAGO trap. Source reduction included collecting discarded containers, scrubbing and rinsing containers that could not be eliminated or treated with larvicide (e.g. animal drinking pans), and sealing cracks and screening vent pipes of septic tanks. Santa Ana went without any vector control from the beginning of the study in May to July 2016, and phase II of the study started when IVC was applied from late July until the end of the study (Fig. 1a). Both SAGO and AGO control traps were serviced every 2 months, which entailed replacing the sticky board, water and hay packet, as well as general cleaning.

We collected and recorded the number of female *Ae. aegypti* per SAGO trap per week from May to November 2016. Data were recorded in the field using iPad Air tablets (Apple Inc., Cupertino, CA, USA) and REDCap software (REDCap Consortium, Nashville, TN, USA). An incident with the computer server on September 8, 2016 caused the loss of data on *Ae. aegypti* abundance from 10 traps in Arcadio and 12 traps in Santa Ana, including the trap located at the center in the latter. Every week, female *Ae. aegypti* specimens collected in SAGO traps were pooled (1–20 specimens/pool) by study site and transported to the Dengue Branch laboratory and stored at -80 °C until tested by real time (TaqMan) RT-PCR assays to detect viral RNA of dengue, chikungunya, and Zika viruses in the body of mosquitoes [14]. RNA of dengue, chikungunya, and Zika viruses can be detected in mosquito specimens exposed in the field on sticky surfaces for over 1 week [13, 15–17]. In order to control for changes in the *Ae. aegypti* populations related to weather, we collected daily temperature, rainfall, and relative humidity data from the nearest weather station in Salinas municipality from May 12 to August 25, then as those data became unavailable, we installed one meteorological station (HOBO Data Loggers, Onset Computer Corporation, Boume, MA, USA) in both areas from September 1 to November 10.

### Statistical analyses

Data are reported as means and standard deviations. We assessed the impact of ICV on the number of female *Ae. aegypti* per trap per week with a generalized linear mixed model analysis (GLMM), with co-variates: treatment (no vector control, vector control), study site (Arcadio, Santa Ana), ring distance (50, 100, 150 m), accumulated rainfall (3rd + 2nd weeks before sampling), average temperature and relative humidity (3 weeks before sampling), and the interaction intervention × ring distance. We used a negative binomial distribution model with log link and first-order autoregressive function for the covariance structure of the repeated measures. Trap ID was included as a random factor to account for trap variability. A posteriori mean comparisons were analyzed using sequential Bonferroni tests at a significance level of 0.05. Statistical analyses were conducted using IBM SPSS Statistics 20 software (IBM Corporation, Armonk, NY, USA).

## Results

Of 179 houses in Arcadio (treatment area) 150 were treated (84%) and 429 control AGO traps were deployed, while in Santa Ana (control area with cross-over treatment after 2 months) 128 of 164 houses were treated (78%) and 361 traps were deployed. Houses that could not be treated were mostly uninhabited or abandoned without ready access. We found the following containers with *Ae. aegypti* immatures: water meters, discarded containers, pails, water barrels, plant pot saucers, plastic pools, trash cans, and water tanks. We observed that 68 of 149 septic tanks in Arcadio were in use (45.6%) and 42 (28.2%) were open, had cracks or their vent pipes lacked mosquito screen. In Santa Ana, 53 of 112 septic tanks were in use (47.3%) and 35 (31.3%) were not mosquito-proof.

**Fig. 2:**
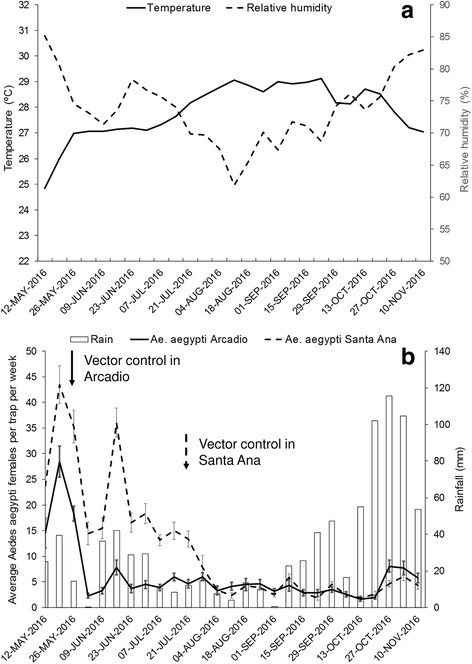
Average and standard error of *Ae. aegypti* females per sentinel AGO trap per week and accumulated rainfall (2–3 weeks before sampling) in Arcadio (**a**) and Santa Ana (**b**) before and after vector control. Rainfall data appear lagged forward 2 weeks to facilitate visual correlations with mosquito density

The GLMM analysis of *Ae. aegypti* females per trap per week was significant (*F*_(8,1102)_ = 147.5, *P* < 0.001), with significant effects of treatment (*F*_(1,1102)_ = 349.7, *P* < 0.001), accumulated rainfall (*F*_(1,1102)_ = 29.1, *P* < 0.001), temperature (*F*_(1,1102)_ = 37.2, *P* < 0. 001), relative humidity (*F*_(1,1102)_ = 12.9, *P* < 0. 001), and the interaction term intervention × ring distance at 50 m (*F*_(4,1102)_ = 2.9, *P* = 0.02). The average number of *Ae. aegypti* females per trap per week in Arcadio changed from 20.4 ± 1.6 before treatment to 4.3 ± 0.2 after treatment (79% reduction; Fig. 2a); in Santa Ana pre- and post-treatment densities were 22.3 ± 1.0 and 3.8 ± 0.2, respectively (83% reduction; Fig. 2b). Increases in *Ae. aegypti* density took place towards the end of the study when rainfall was abundant, although these increases were small when compared with pre-treatment periods (Figs. 2a, b).

**Fig. 3:**
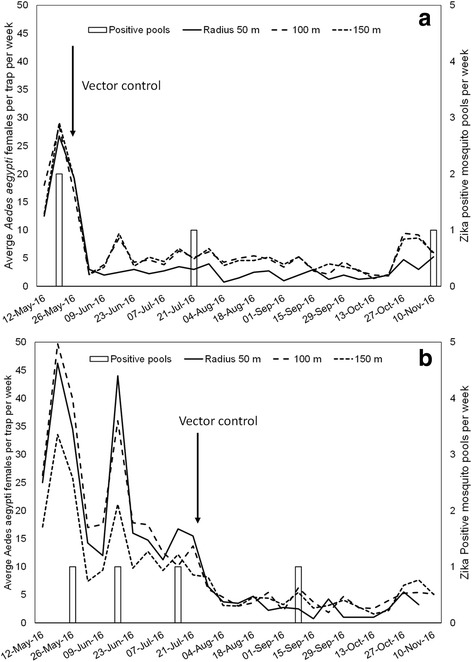
Number of female *Ae. aegypti* per sentinel AGO trap per week captured within the inner (50 m), middle (100 m), and outer rings (150 m) in Arcadio (**a**) and Santa Ana (**b**), before and after vector control

**Fig. 4:**
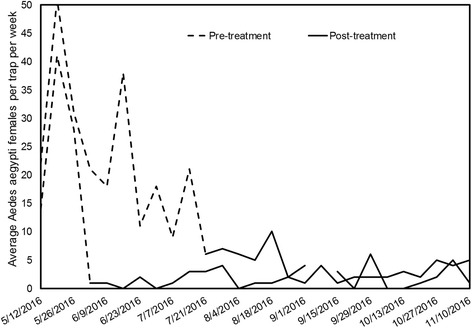
Number of female *Ae. aegypti* per sentinel AGO trap per week captured at the target house before and after vector control from May to November 2016 in Arcadio and Santa Ana, Salinas, Puerto Rico

Overall, average mosquito densities before treatment for both areas were similar in the inner (15.6 ± 2.8), intermediate (18.1 ± 1.2), and outer rings (15.6 ± 1.7; Fig. 3). After treatment, densities significantly decreased (*post-hoc* tests, each significant at *P* < 0.05, test statistics not shown) to 2.8 ± 0.5, 4.1 ± 0.4, and 4.3 ± 0.4 mosquitoes per trap per week in the inner, middle, and outer rings, respectively. The GLMM significant interaction term results from the lower density of mosquitoes captured within the 50 m ring (Fig. 3). Mosquito density at the target house in Arcadio changed from 27.7 ± 7.8 before intervention to 2.1 ± 0.3 after intervention (92.4% reduction), whereas in Santa Ana pre- and post-treatment densities were 22.4 ± 4.0 and 3.5 ± 0.8, respectively (84.3% reduction) (Fig. 4). Thus, overall mosquito density at the target houses in both areas (2.6 ± 0.4) was similar to the average density observed in the 50 m rings (2.8 ± 0.5) after vector control.

We found four Zika positive mosquito pools in Arcadio out of 180 processed pools (3437 females): two in May, one in July, and one in November 2016, and four in Santa Ana out of 326 processed pools (6407 females): one each in May, June, July, and September 2016 (Fig. 3). We did not detect any positive mosquito pools for dengue or chikungunya viruses in the study sites.

## Discussion

This investigation showed that applying IVC to non-isolated urban areas with 150 m radii reduced the population of gravid females of *Ae. aegypti* to or below an empirically pre-defined mosquito density threshold of three females per trap per week at and around a target house in the center of the treated areas. Moreover, the observed reduction was sustained throughout the study after applying vector control measures and servicing the traps every 2 months. The experimental design was conducted in two phases: the first consisted of pre-post treatment observations, a contemporaneous control to compare with a treatment area, and the second phase was a partial cross-over design, where IVC was applied to the original untreated area (Santa Ana) after 2 months of observations to determine if *Ae. aegypti* density converged to the levels observed in the original treatment area (Arcadio). As observed in a previous study in the same municipality of Puerto Rico using the same IVC method but in relatively isolated areas [18], the density of *Ae. aegypti* recorded in the originally untreated area after IVC did converge to the lower and steady densities observed in the originally treated area. The reduction of female *Ae. aegypti* was not as large in the middle (50–100 m; 4.1 mosquitoes/trap/week) and outer (100–150 m; 4.3) rings as in the inner (0–50 m; 2.8) ring, but was nevertheless significant and close to the defined mosquito density threshold. Assuming that vector control was uniform through the 150 m areas, the lower mosquito density in the inner ring was possibly the result of a limited dispersal of *Ae. aegypti*.

Creating a protecting zone around a target house, such as at a pregnant woman’s house, may significantly protect against *Ae. aegypti*-borne viruses. However, because people including pregnant women are mobile, protection at home is likely to be partial; that is, people can become infected elsewhere. Recent studies of spatial and temporal dispersal of dengue virus lineages in Thailand suggested that human cases living within 200 m of each other had more than 80% chance of sharing a common virus ancestor and 60% of case pairs came from the same transmission chain, evidencing that most infections were locally acquired [19]. Studies of dengue case contact-tracing conducted in Cairns, Australia to determine spatial and temporal patterns of transmission showed that 43% of cases were directly linked to the home of patients [20]. Because the transmission of these viruses can occur at the household level, a combination of a protective halo along with other actions such as screens in windows and doors, spatial repellents, topical repellents, wearing appropriate clothing, and avoiding the production of mosquitoes around homes may together significantly contribute to overall protection.

Targeted indoor residual spraying of insecticides has been proposed to prevent the spread or recurrence of dengue virus infections at sprayed homes [20, 21]. However, *Ae. aegypti* has been found to be resistant or partially resistant to eight pyrethroids used for residual or spatial spray applications in Puerto Rico [22] (CDC, unpublished). Previous *Ae. aegypti* control efforts using source reduction and larviciding in a rural area in Puerto Rico were successful at reducing the density of mosquito pupae in superficial containers, but failed to reduce the number of adult mosquitoes because of the presence of highly productive, underground cryptic aquatic habitats [23]. Thus, advancing *Ae. aegypti* control will be facilitated by exploring alternative, non-insecticidal vector control tools targeting the adult mosquitoes. For example, Mains et al. [24] released 182,000 *Wolbachia* bacteria-infected males of *Aedes albopictus* from June to September 2014 in an area with a 250 m radius in suburban Lexington, Kentucky to reduce the local population of this mosquito species. Cytoplasmic incompatibility and sterility occur when *Wolbachia-*infected males mate with mosquito females that lack the same *Wolbachia* strain [25]. Such an approach has the potential for use, as in this study, to protect targeted houses. A much-needed step further regarding the effectiveness of vector control strategies is demonstrating impact on epidemiologic indicators [12] or on replicable entomological indicators, such as potential threshold mosquito densities below which local transmission does not occur. The importance of having a reliable target for *Ae. aegypti* control lies in the simplification of experimental designs and reduction of costs, because the proof of effectiveness does not need to come from comparisons between treated and untreated areas, but whether mosquito density in the treated area falls below the threshold. Having a threshold would also simplify sampling because more efficient sampling techniques could be used to estimate mosquito density, such as sequential sampling [26].

While ZIKV virus was detected in both buffer areas, we were not able to use the presence of Zika virus in mosquitoes as a proxy for the impact of vector control in this study (e.g. before *vs* after treatment, untreated *vs * treated areas) because positive pools were infrequently detected. Outbreaks of Zika virus in *Ae. aegypti* were observed in concurrent studies in other neighbourhoods in Salinas municipality in March–April 2016 and later in July–September 2016 (CDC, unpublished). Zika virus thus appears to have passed through this neighbourhood prior to the start of the study in May 2016. Mosquito densities observed before the interventions in both buffer areas were substantial and at or above densities observed in areas where both chikungunya and Zika viruses were extensively detected in *Ae. aegypti* (CDC, unpublished). Because *Ae. aegypti* mosquitos do not move far on their own [9, 10], the presence of virus in local mosquitoes reflects the presence of local infectious people and thus can serve as a surveillance tool for local transmission [13].

## Conclusions

Our results demonstrate that the density of female *Ae. aegypti* can be significantly reduced at a target house in the center of a circular area with a 150 m radius by integrating source reduction, larviciding, and mass trapping. Mosquito density after IVC was the lowest in the center of the areas, possibly reflecting limited dispersal of *Ae. aegypti* from adjacent areas. We evaluated the impact of mosquito reduction by the capacity of this vector control strategy to bring and keep down female mosquito populations below an empirically pre-defined threshold of three specimens per trap per week, a measure correlated with reduced arboviral diseases incidence.

## Abbreviations

AGO: Autocidal gravid traps
ICV: Integrated vector control
SAGO: Sentinel autocidal gravid traps
